# Carbapenem resistance in extensively drug-resistant *Salmonella enterica* serovar Agona and AmpC β-lactamase-producing* S.* Infantis

**DOI:** 10.1128/spectrum.02922-23

**Published:** 2023-10-03

**Authors:** Ping-Chun Hsu, You-Wun Wang, Bo-Han Chen, Yu-Ping Hong, Ru-Hsiou Teng, Po-Yu Liu, Chien-Shun Chiou

**Affiliations:** 1 Division of Infectious Diseases, Department of Internal Medicine, Taichung Veterans General Hospital, Taichung, Taiwan; 2 Center for Diagnostics and Vaccine Development, Centers for Disease Control, Taichung, Taiwan; University of Pittsburgh, Pittsburgh, Pennsylvania, USA

**Keywords:** Salmonellosis, non-typhoidal salmonella (NTS), antimicrobial resistance, extensively drug-resistant (XDR), carbapenem resistance, whole-genome sequencing, transposon

## Abstract

We report that carbapenem resistance developed in an extensively drug-resistant *Salmonella enterica* serovar Agona strain during antimicrobial therapy and in an AmpC β-lactamase-producing *S*. Infantis strain. Whole-genome sequence analysis indicated that carbapenem resistance in the *S*. Agona strain was associated with the insertion of a 160-kb IS15DI composite transposon in *ompC_378*, which encodes a porin consisting of 378 amino acids, along with a 397-bp deletion in the *ompD*. The transposon harbored 12 resistance genes including *bla*_CTX-M-55_ and an efflux pump regulatory gene, *ramAp*. Carbapenem resistance in the *bla*_CMY-2_-carrying *S*. Infantis strain was linked to the insertion of a 22,905-bp segment located 10 bp upstream of the *ompC_378* coding sequence, as well as a 254-bp deletion in *ompD*. Our findings indicate that the loss of both *ompC_378* and *ompD* is necessary for carbapenem resistance in extended-spectrum β-lactamase and AmpC β-lactamase-producing *Salmonella* strains.

**IMPORTANCE:**

Carbapenem resistance arising from the loss of porins is commonly observed in extended-spectrum β-lactamase (ESBL) and AmpC β-lactamase-producing strains of certain *Enterobacteriaceae* genera, including *Klebsiella pneumoniae*, *Escherichia coli*, and *Pseudomonas aeruginosa*. However, this resistance mechanism is rarely reported in the *Salmonella* genus. To address this knowledge gap, our study offers genetic evidence demonstrating that the loss of two specific porins (OmpC_378 and OmpD) is crucial for the development of carbapenem resistance in *Salmonella* ESBL and AmpC β-lactamase-producing strains. Furthermore, our findings reveal that most *Salmonella* serovars carry seven porin parathologs, with OmpC_378 and OmpD being the key porins involved in the development of carbapenem resistance in *Salmonella* strains.

## INTRODUCTION

*Salmonella* is a major cause of gastroenteritis worldwide. Non-typhoidal *Salmonella* (NTS) was estimated to cause 93.8 million gastroenteritis globally and 155,000 deaths in 2006 (1). While NTS usually causes self-limiting gastroenteritis, invasive NTS infections often result in significant mortality rates. In 2017, NTS was estimated to cause 535,000 invasive infections and 77,500 deaths worldwide (2). Previously, chloramphenicol, amoxicillin (ampicillin), and cotrimoxazole (trimethoprim/sulfamethoxazole) were the primary drugs used to treat invasive salmonellosis. However, due to the prevalence of resistance to these first-line drugs, extended-spectrum cephalosporins (e.g., ceftriaxone) and fluoroquinolones (e.g., ciprofloxacin) have been recommended as alternative treatment options (3). Unfortunately, the extensive use of second-line antimicrobials has led to the emergence of resistance to the extended-spectrum cephalosporins and fluoroquinolones in NTS strains, posing a concerning challenge in the medical management of infections (3). Carbapenems and azithromycin are now considered a last-resort treatment option for invasive *Salmonella* infections caused by multidrug and extensively drug-resistant strains (3, 4).

Although carbapenem resistance remains rare in NTS, isolates displaying such resistance have been identified in humans, companion animals, livestock, wild animals, and food (5). The resistance primarily develops through the acquisition of carbapenemase genes or porin loss combined with the production of AmpC-type β-lactamase or extended-spectrum β-lactamase (ESBL) (5). While the development of carbapenem resistance during therapy has been observed in various bacterial infections (6 – 9), its occurrence in *Salmonella* has been rarely reported (10, 11).

In this study, we present a case of carbapenem resistance developed in *S. enterica* serovar Agona during therapy, as well as the resistance in an *S*. Infantis strain. We performed whole-genome sequence analysis to decipher the genetic characteristics and the mechanism of carbapenem resistance in the two resistant strains.

## RESULTS

### Genetic characteristics of *S*. Agona strains

Two *Salmonella* isolates R21.2429 and R21.2430 recovered from a patient during therapy were identified to be serovar Agona by PFGE pattern comparison (12) and *in silico* whole-genome sequence-based typing (SISTR). They were determined to be ST13 and harbored 14 resistance genes: *aac(3)-IId*, *aac(6′)-Iaa*, *aadA22*, *aph(3′)-Ia*, *arr-2*, *bla*_CTX-M-55_, *bla*_TEM-1B_, *dfrA14*, *floR*, *fosA7*, *lnu(F*), *qnrS13*, *sul3*, and *tet(A),* an efflux pump regulatory gene, *ramAp*, and T57S mutation in parC. No plasmids were found in the two isolates.

The sizes of the chromosomes in *S*. Agona R21.2429 and R21.2430 were determined to be 5,023,197 bp and 5,020,666 bp, respectively. Except for *aac(6′)-Iaa* and *fosA7*, the 12 other resistance genes and *ramAp* were found within a 140,640-bp genomic island. This genomic island was flanked by two IS15DI elements, forming an IS15DI composite transposon that was inserted in an *ompC*, named *ompC_378*, a gene encoding a porin consisting of 378 amino acids. IS15DI belongs to the IS6 family and differs from IS26 by 3 bp (13). The IS15DI composite transposon shared a large part of the sequence with a plasmid named pR18.0877_278k (GenBank accession CP037959.1). This plasmid, having IncHI2-IncHI2A replicons and a size of 278 kb, was initially identified in an XDR *S*. Goldcoast strain (14). pR18.0877_278k harbored 15 resistance genes and a *ramAp*, all located within a 160,060-bp DNA segment flanked by two IS640 elements, forming an IS640 composite transposon.

**Fig 1:**
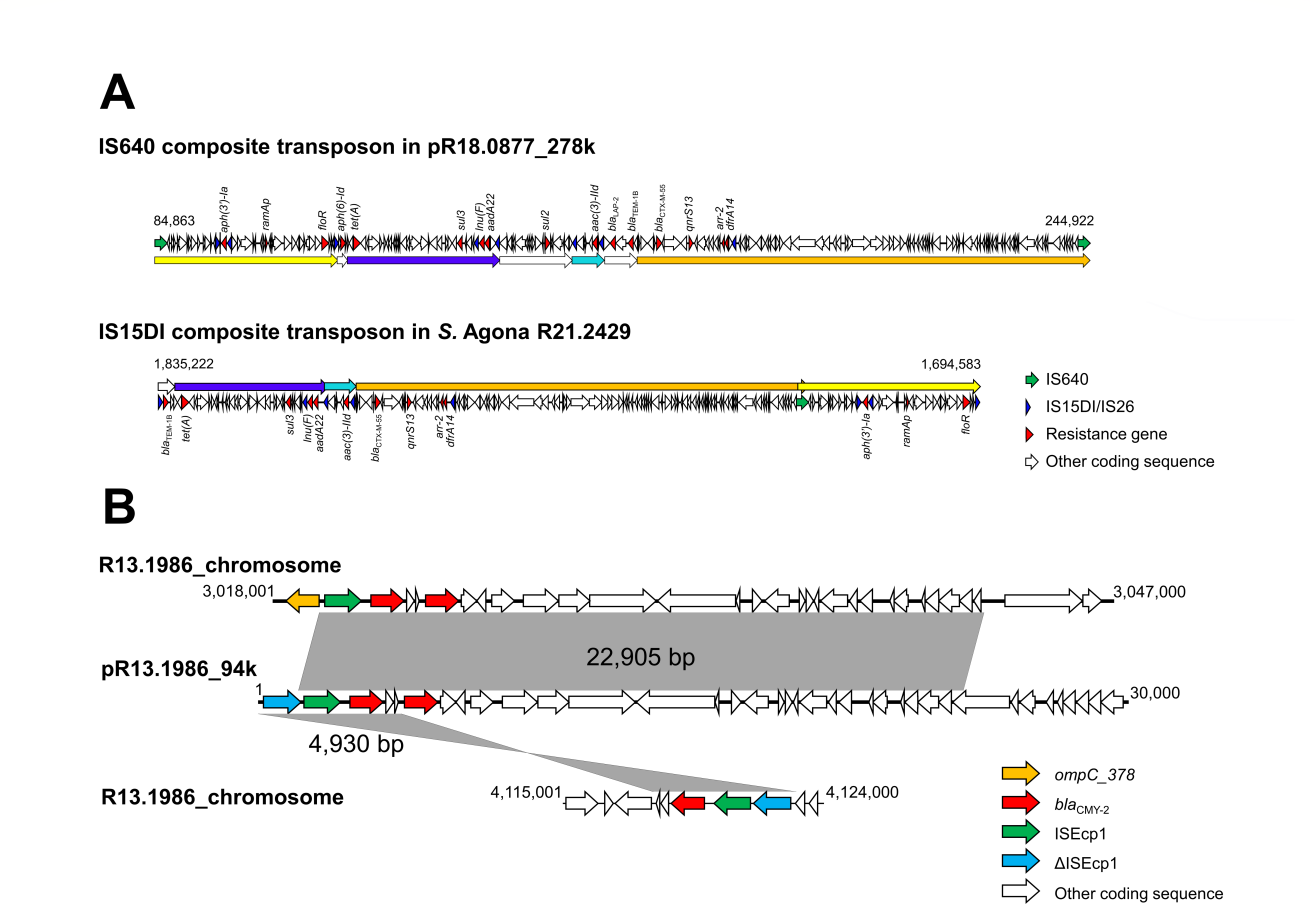
(**A**) Schematic map of IS640 composite transposon in plasmid pR18.0877_278k (GenBank accession no. CP037959.1) and IS15DI composite transposon in the chromosome of *S*. Agona R21.2429 (accession no. CP090929.1); (**B**) ISEcp1- *bla*_CMY-2_-harboring segments in the chromosome and plasmid pR13.1986_94k of *S*. Infantis strain R13.1986 (accession no. CP129384.1 and CP129386.1).

The IS15DI composite transposon found in the two *S*. Agona isolates exhibited significant similarity to the IS640 composite transposon (Fig. 1A). However, the IS640 composite transposon contained three DNA segments housing a total of four resistance genes (*aph (6)-Id*, *bla*_TEM-1B_, *bla*_LAP-2_, and *sul2*), which were absent in the IS15DI composite transposon. Conversely, a 3,021-bp DNA segment of the IS15DI transposon, containing *bla*_TEM-1B_, was not present in the IS640 composite transposon. Both transposons shared 12 common resistance genes and a *ramAp*. In terms of chromosomal variations, R21.2429 contained an additional IS640-like element (2,130 bp) and a 4-bp tandem repeat resulting from the transposition of the insertion sequence. On the other hand, R21.2430 had a 397-bp deletion in *ompD*.

### Genetic characteristics of carbapenem-resistant *S*. Infantis R13.1986

The *S*. Infantis strain was identified to be ST32. It had a chromosome size of 4,7112,862 bp and contained six plasmids: pR13.1986_108k (belonging to the IncFII incompatibility group, 108,036 bp in size), pR13.1986_94k (IncI1-I(α), 94,398 bp), pR13.1986_58k (IncL, 58,960 bp), pR13.1986_50k (IncFIC, 50,239 bp), pR13.1986_4k (Col440II, 4,779 bp), and pR13.1986_3k (ColRNAI-like, 3,205 bp). The strain carried five copies of *bla*_CMY-2_, with three copies located in the chromosome and two copies present in pR13.1986_94k. Our genomic sequence analysis identified a 22,905-bp segment that contained an ISEcp1 and two copies of *bla*_CMY-2_ and was inserted 10 bp upstream of the coding sequence of *ompC_378*. This 22,905 bp segment was also present in pR13.1986_94 k (Fig. 1B). In addition, another 4,930 bp segment carrying an ISEcp1, a defective ISEcp1, and a *bla*_CMY-2_, was found inserted elsewhere in the chromosome (Fig. 1B). The transposition of these *bla*_CMY-2_-carrying segments is likely mediated by ISEcp1 through a one-ended transposition process in that only a single IS element is involved (15). As a result of ISEcp1-mediated transposition, a 5-bp direct repeat was generated at the insertion sites. Furthermore, our sequence analysis also found a 254-bp deletion within *ompD*.

### Antimicrobial susceptibility

Compared to the pan-susceptible *S*. Agona strain R17.4368, both XDR *S*. Agona strains (R21.2429 and R21.2430) showed resistance or reduced susceptibility to 14 antimicrobials, as listed in Table 1. The resistance to ampicillin, cefotaxime, ceftazidime, ceftriaxone, cefepime, chloramphenicol, gentamicin, sulfamethoxazole, trimethoprim, and tetracycline was attributed to the acquired resistance genes carried by the two strains (Table 1). The resistance to azithromycin, nalidixic acid, and tigecycline was likely contributed by *ramAp* through activating the expression of multidrug efflux pumps such as AcrAB-TolC (16). The resistance to ciprofloxacin was due to *qnrS13* and *ramAp*, as Qnr genes alone typically confer only low-level resistance to fluoroquinolones in *Enterobacteriaceae* (17). The ciprofloxacin resistance was not linked to ParC T57S substitution, as this substitution is known not to play a role in ciprofloxacin resistance in *Salmonella* (18). In comparison to R17.4368, R21.2429 showed reduced susceptibility to ampicillin/sulbactam and cefoperazone/sulbactam, which could be associated with *ramAp*, as the β-lactamase inhibitors in these combinations could serve as substrates for multidrug efflux pumps activated by RamAp (19).

**TABLE 1:** Antimicrobial susceptibility and resistance determinants in *Salmonella* strains

Antimicrobial	Susceptibility (MIC, mg/L)^*b*^				Resistance in *S*. Agona strains linked to:	
	*S*. Agona R17.4368	*S*. Agona R21.2429	*S*. Agona R21.2430	*S*. Infantis R13.1986	Resistance gene	Δ*ompCD^c^*
Azithromycin	S (16)	R (64)	R (64)	S (16)	*ramAp*	No
Ampicillin	S (2)	R (>64)	R (>64)	R (>64)	*bla*_TEM-1B_, *bla*_CTX-M-55_	ND
Ampicillin/sulbactam^*a*^	S (≤2)	I (16)	R (>16)	R (>16)	*ramAp*	Yes
Cefoperazone/sulbactam^*a*^	S (≤8)	S (16)	R (>32)	R (>32)	*ramAp*	Yes
Cefotaxime	S (≤0.25)	R (>4)	R (>4)	R (>4)	*bla*_CTX-M-55_	ND
Ceftazidime	S (0.5)	R (>8)	R (>8)	R (>8)	*bla*_CTX-M-55_	ND
Ceftriaxone^*a*^	S (≤0.25)	R (>32)	R (>32)	R (>32)	*bla*_CTX-M-55_	ND
Cefepime^*a*^	S (≤0.12)	R (16)	R (>16)	R (16)	*bla*_CTX-M-55_	Yes
Flomoxef^*a*^	S (≤2)	S (≤2)	I (32)	R (>32)		Yes
Ertapenem^*a*^	S (≤0.12)	S (≤0.12)	R (>4)	R (>4)		Yes
Imipenem*^a^*	S (≤0.25)	S (≤0.25)	S (≤0.25)	R (8)		No
Meropenem	S (0.06)	S (0.06)	I (2)	R (8)		Yes
Colistin	S (2)	S (≤1)	S (≤1)	S (1)		No
Nalidixic acid	S (8)	R (32)	R (32)	S (8)	*ramAp*	No
Ciprofloxacin	S (0.03)	R (2)	R (4)	S (0.06)	*qnrS13, ramAp*	Yes
Gentamicin	S (1)	R (>32)	R (>32)	S (1)	*aac (3)-IId*	ND
Chloramphenicol	I (16)	R (>128)	R (>128)	I (16)	*floR, ramAp*	ND
Sulfamethoxazole	S (128)	R (>1,024)	R (>1,024)	S (16)	*sul3, ramAp*	ND
Trimethoprim	S (0.5)	R (>32)	R (>32)	S (0.25)	*dfrA14, ramAp*	ND
Tetracycline	S (4)	R (>64)	R (>64)	S (4)	*tet(A), ramAp*	ND
Tigecycline	S (0.5)	R (4)	R (4)	S (0.5)	*ramAp*	No

^*a*^ MIC was measured using the VITEK 2 system.

^*b*^ S, susceptible; I, intermediate; R, resistance.

^*c*^ ND, not determined; No, no observable effect on susceptibility; Yes, elevated resistance.

When compared to R21.2429, R21.2430 exhibited higher minimum inhibitory concentrations (MICs) for seven antimicrobials, including ampicillin/sulbactam, cefoperazone/sulbactam, cefepime, flomoxef, ertapenem, meropenem, and ciprofloxacin. Genomic sequence analysis of the two isolates revealed that R21.2430 had an additional 397 bp deletion in *ompD*. The defects in *ompC_378* and *ompD* were associated with elevated MICs for the seven antimicrobials but did not significantly affect susceptibility to imipenem, colistin, nalidixic acid, and tigecycline (Table 1). Regarding the three carbapenems, the deficiency of OmpC_378 and OmpD in *S*. Agona R21.2430 had a greater impact on resistance to ertapenem compared to meropenem, but it had no observable effect on susceptibility to imipenem. However, in the *bla*_CMY-2_-carrying *S*. Infantis R13.1986, the deficiency of OmpC_378 and OmpD had a significant effect on resistance to ertapenem, meropenem, and imipenem (Table 1). In addition, *S*. Infantis R13.1986 displayed resistance to all β-lactam drugs and two β-lactamase inhibitor/β-lactam combinations tested, which was primarily attributed to the carriage of *bla*_CMY-2_, an AmpC β-lactamase gene.

### Porins associated with carbapenem resistance

**Fig 2:**
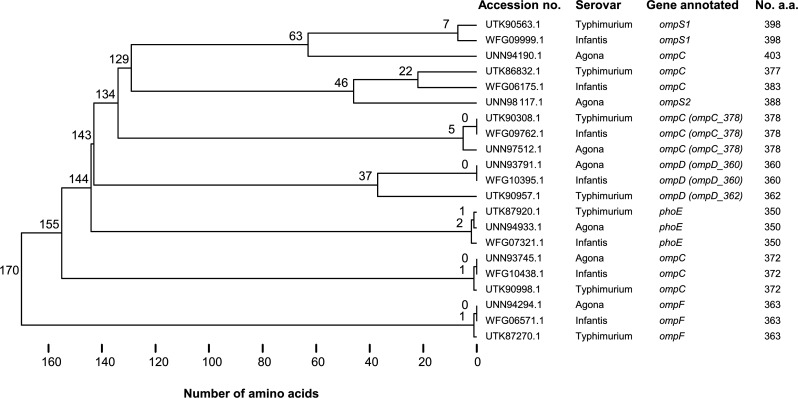
Differences in amino acid sequences of porins found in *S*. Agona R21.0464 (accession no. CP093402.1), *S*. Infantis R21.0914 (CP121066.1), and *S*. Typhimurium R18.0292 (CP100739.1).

The OmpD sequence from *S*. Agona R21.2429 shared 56% to 100% sequence identity with seven porins in each of the strains *S*. Agona R21.0464, *S*. Infantis R21.0914, and *S*. Typhimurium R18.0292. These porin sequences differed from each other by 0 to 170 amino acids and were annotated to be OmpC, OmpD, OmpF, OmpS1, OmpS2, and phosphoporin PhoE (Fig. 2). Among the seven porin genes identified in each strain, three were assigned to *ompC* and one to *ompD*. Notably, *ompD* and an *ompC*, which encodes a porin of 378 amino acids (denoted as *ompC_378*), were all defective in the carbapenem-resistant strains *S*. Agona R21.2430 and *S*. Infantis R13.1986, as well as the previously reported *S*. Typhimurium S4 (10).

Further investigation by blasting an OmpD sequence against *Salmonella* genomes in the NCBI database found two distinct types or clusters of OmpD, namely OmpD_360 (consisting of 360 amino acids) and OmpD_362 (362 amino acids) (Fig. 2). The two OmpD types exhibited a distinct distribution pattern and serovar preference. Specifically, *ompD_360* was identified in nearly all genomes of serovars Agona, Infantis, and Senftenberg, whereas *ompD_362* was found in almost all genomes of serovars Enteritidis, Typhimurium, Newport, and Heidelberg (data not shown). However, both types were more evenly distributed in serovars Anatum and Kentucky. Notably, *ompD* was not identified in any *S*. Typhi genomes, and it was absent in approximately half of the *S*. Dublin and *S*. Reading genomes.

## DISCUSSION

We present the first documented case of the emergence of carbapenem resistance during antimicrobial therapy in a *S*. Agona infection. In addition, we investigate the resistance mechanisms in both carbapenem-resistant strains of *S*. Agona and *S*. Infantis. The *S*. Infant strain is the only one observed exhibiting carbapenem resistance among 25,663 clinical *Salmonella* isolates recovered in Taiwan from 2004 to 2021. The development of carbapenem resistance during therapy is uncommon in *Salmonella* species. A previous report by Armand-Lefèvre et al. described that the emergence of imipenem resistance in an *S*. Wien strain during antimicrobial therapy was attributed to CMY-4 β-lactamase production and porin loss (11). Another report by Su et al. demonstrated that the development of ertapenem and imipenem resistance in an *ompD*-deficient *S*. Typhimurium strain was linked to CMY-2 β-lactamase production and subsequent loss of OmpC (10). In contrast, the emergence of carbapenem resistance during therapy is relatively common in organisms such as *Klebsiella pneumoniae* (20, 21), *Pseudomonas aeruginosa* (9, 22), *Escherichia coli* (6, 23), and *Enterobacter* spp (7). In these organisms, carbapenem resistance is primarily acquired through porin loss, but less commonly via the acquisition of carbapenemase genes (6, 9). In our study, we demonstrate that the development of ertapenem resistance in an *ompC*-deficient *S*. Agona strain is linked to the presence of an ESBL gene, *bla*_CTX-M-55_, and further loss of *ompD*. Furthermore, carbapenem resistance in the *S*. Infantis strain is associated with the presence of an AmpC gene, *bla*_CMY-2_, accompanied by the loss of *ompC* and *ompD*.

Our data indicate that the *S*. Agona strain lacking *ompC_378* and *ompD* expression exhibited resistance to ertapenem and reduced susceptibility to meropenem, while its susceptibility to imipenem remained unchanged (Table 1). In contrast, imipenem resistance was found in a porin-deficient *S*. Wien strain and a porin-deficient *S*. Typhimurium strain (10, 11). These variations in carbapenem resistance profiles could potentially be attributed to the presence of different classes of β-lactamases. In a study by Jacoby et al., the susceptibility in an Omp35- and Omp36-deficient *K. pneumoniae* strain to seven β-lactams including ertapenem, meropenem, and imipenem was investigated with various β-lactamase genes (24). The results indicated that most porin-deficient strains carrying an AmpC β-lactamase gene exhibited higher MICs for ertapenem, imipenem, and meropenem compared to those carrying an ESBL gene. Our study further demonstrates that the resistance to ertapenem, imipenem, and meropenem in *S*. Infantis R13.1986 was linked to the carriage of *bla*_CMY-2_ and the deficiency of *ompC* and *ompD*. Therefore, it can be inferred that *Salmonella* strains with defective OmpC and OmpD, while harboring an AmpC β-lactamase gene, are likely to exhibit resistance to ertapenem, imipenem, and meropenem. Conversely, porin-deficient *Salmonella* strains carrying an ESBL gene are likely to be resistant only to ertapenem but not imipenem.

Many studies have highlighted the diverse impacts of outer membrane porins on antimicrobials across various bacteria. A study conducted by Tsai et al. indicates that the deletion of *ompK35* (comparable to *ompC* in *Salmonella*) in *K. pneumoniae* did not result in a significant change in the MICs of cephazolin, cephalothin, and cefoxitin. However, the deletion of *ompK36* (equivalent to *ompD* in *Salmonella*) caused an increase in MICs for these three antimicrobials, shifting them from susceptible to resistant (25). The study further revealed that the simultaneous deletion of *ompK35* and *ompK36* had a more significant impact on antimicrobial susceptibility, resulting in 8- and 16-fold increases in the MICs of meropenem and cefepime. Another study conducted by Choi and Lee explored the effects of single, double, and triple porin deletions in *E. coli*, demonstrating distinct impacts on antimicrobial susceptibility. Specifically, double deletion of *ompC* and *ompF* (equivalent to *ompD* in *Salmonella*) led to elevated MICs for all the tested β-lactams, including meropenem, but not for imipenem (26). In a study on *Salmonella*, Chowdhury et al. reported that single deletions of *ompC*, *ompD*, or *ompF* did not confer protection to *Salmonella* against ceftazidime and meropenem (27).

Our study findings demonstrate that the *ompC*-defective *S*. Agona strain (R21.2429) did not significantly affect the MICs of ertapenem, meropenem, and imipenem. However, the *ompC_378* and *ompD*-defective *S*. Agona strain (R21.2430) exhibited elevated MICs for ertapenem and meropenem, although susceptibility to imipenem remained unchanged (Table 1). In another case, Su et al. reported that *ompD* deficiency in a *bla*_CMY-2_-carrying *S*. Typhimurium strain did not result in carbapenem resistance, but resistance emerged after the further loss of OmpC_378 in the strain. Thus, these two cases indicate that the loss of both OmpC_378 and OmpD functionality in ESBL- or AmpC β-lactamases-producing strains is required for carbapenem resistance in *Salmonella*. Furthermore, the carbapenem-resistant *S*. Infantis strain characterized in our study also exhibited deficiencies in OmpC_378 and OmpD. Nevertheless, it is noteworthy that *Salmonella* strains producing ESBL- or AmpC β-lactamases, along with a defective *ompC* or *ompD*, may be more prone to developing carbapenem resistance during therapy.

During our investigation of *ompD* sequences in three strains of *S*. Agona, *S*. Infantis, and *S*. Typhimurium, we identified seven porin genes in each genome, exhibiting more than 50% amino acid sequence identity among them. Among the seven genes, three are annotated as *ompC*. Subsequent analysis revealed that the *ompC* encoding 378 amino acids (referred to as *ompC_378*) was defective in all carbapenem-resistant strains *S*. Agona R21.2430, *S*. Infantis R13.1986, and the *S*. Typhimurium S4 that was previously described by Su et al. (10). Further investigation through blasting an OmpC_378 sequence with the *Salmonella* genomes in the NCBI database indicates that this gene is considerably conserved among *Salmonella* serovars (data not shown). In addition, we observed two distinct types of OmpD, with each displaying a distinct distribution pattern and serovar preference. For instance, OmpD_360 is present in almost all serovars Agona and Infantis, whereas OmpD_362 is carried in nearly all serovars Enteritidis and Typhimurium. It is worth noting that *S*. Typhi lacks the *ompD* gene. This raises the question of whether strains of this serovar can develop carbapenem resistance through the loss of other porins of the family. Further investigations are needed to explore the role of other porins in resistance to antimicrobials, particularly carbapenems.

The XDR *S*. Agona strains investigated in this study exhibit resistance to all clinically recommended antimicrobials for salmonellosis treatment, including ampicillin, chloramphenicol, cotrimoxazole, extended-spectrum cephalosporins (e.g., ceftriaxone), fluoroquinolones (e.g., ciprofloxacin), and azithromycin. In cases of XDR *Salmonella*, meropenem and azithromycin are considered the last-resort treatment option (4). However, meropenem alone may not effectively treat invasive *Salmonella* infections (28), particularly those caused by AmpC β-lactamase-producing *Salmonella* strains with porin deficiencies. In this case, we administered ceftazidime/avibactam, a novel cephalosporin/β-lactamase inhibitor combination agent, to treat the patient. It should be noted that, to the best of our knowledge, ceftazidime/avibactam is not currently established as the standard treatment for salmonellosis. Further investigations are necessary to determine the optimal therapy for XDR *Salmonella* infections, especially given the increasing prevalence of XDR *Salmonella*, such as *S.* Goldcoast, in Taiwan since 2018 (14). Through the analysis of whole-genome sequences of the isolates, we identified the resistance determinants in the XDR *Salmonella* strains. This information on resistance determinants can aid healthcare professionals in identifying additional alternative drugs that can effectively combat *Salmonella* strains.

In conclusion, our study reveals that defects in both OmpC_378 and OmpD are necessary for carbapenem resistance in *Salmonella* strains producing AmpC or ESBL β-lactamases. These findings shed light on the mechanisms underlying carbapenem resistance in *Salmonella* and have significant implications for surveillance and treatment strategies in combating antimicrobial resistance.

## MATERIALS AND METHODS

### The case with carbapenem-resistant *S*. Agona infection

An 83-year-old woman with type 2 diabetes mellitus was admitted to the Taichung Veterans General Hospital in Taiwan in 2020 due to fever, chills, and dyspnea. The patient had been in her usual state of health until 1 week before the admission. Upon admission, she was initially treated with empiric piperacillin/tazobactam. A stool culture revealed the presence of serogroup B *Salmonella*. The isolate, designated as R21.2429, was identified using MALDI-TOF (bioMérieux, Inc.) and BD Difco *Salmonella* antisera (BD, Inc.). Antimicrobial susceptibility testing of R21.2429 conducted using the VITEK 2 system indicated resistance to the third- and fourth-generation cephalosporins (ceftazidime, ceftriaxone, and cefepime), trimethoprim/sulfamethoxazole, and ciprofloxacin, while susceptibility to ertapenem and imipenem was observed. Consequently, the patient’s antimicrobial therapy was switched to ertapenem. However, after 7 days of ertapenem therapy, the patient continued to experience diarrhea, and a subsequent stool culture yielded *Salmonella* of serogroup B. An isolate, designated as R21.2430, was saved for further analysis. Notably, in addition to the resistance observed in R21.2429, R21.2430 developed resistance to ertapenem. Consequently, ceftazidime/avibactam therapy was initiated. After 12 days of treatment with ceftazidime/avibactam, the patient’s symptoms and signs resolved, leading to her discharge. A follow-up stool culture did not detect the presence of *Salmonella*.

### *S*. Agona isolates

The serogroups of *Salmonella* isolates R21.2429 and R21.2430, recovered from the patient, as mentioned above, were determined using the Denka Seiken Agglutinating Sera *Salmonella* Antisera Sets (Denka Seiken, Japan). The serotypes were determined by comparing PFGE patterns with those in the *Salmonella* Fingerprint database of the Taiwan Centers for Disease Control (Taiwan CDC) (12), and later by whole-genome sequences using the tool SISTR (https://github.com/phac-nml/sistr_cmd) (29). A pan-susceptible *S*. Agona strain R17.4368 was included for the comparison of antimicrobial susceptibility. R17.4368 was recovered from a salmonellosis patient in Taiwan in 2017; it exhibited a PFGE pattern highly similar to those of R21.2429 and R21.2430 and contained only a resistance gene, *fosA7*, in the chromosome.

### Carbapenem-resistant *S*. Infantis isolate

Carbapenem resistance was detected in *Salmonella* isolates obtained from various hospitals across Taiwan between 2004 and 2021, as part of the PulseNet Taiwan disease surveillance project. During this period, a total of 38,957 *Salmonella* isolates were collected, and antimicrobial susceptibility testing was performed on 25,663 isolates. From these isolates, only one, belonging to the serovar Infantis, displayed carbapenem resistance.

### Antimicrobial susceptibility testing

Susceptibility testing was performed at the Taichung Veterans General Hospital using the *VITEK 2* system (bioMérieux, Inc.) and at the Central Region Laboratory of Taiwan CDC using the Thermo Scientific Sensititre EUVSEC MIC panels to determine the MICs of ampicillin, azithromycin, cefotaxime, ceftazidime, chloramphenicol, ciprofloxacin, colistin, gentamicin, meropenem, nalidixic acid, sulfamethoxazole, tetracycline, tigecycline, and trimethoprim. The interpretation of MIC results primarily followed the CLSI breakpoints (30), with secondary reference to the EUCAST breakpoint tables (31), or the breakpoints recommended by the manufacturer of the testing panels.

### Genetic analysis

Bacterial isolates were subjected to PFGE analysis using the PulseNet protocol (32) and whole-genome sequencing. For whole-genome sequencing, both short sequence reads and long sequence reads were generated using the Illumina Miseq System (Illumina, Inc.) and the Oxford Nanopore MinION sequencing platform (Oxford Nanopore Technologies, Inc.), respectively. The reads generated from the Illumina Miseq System were assembled using the SPAdes assembler (33). The assembled contigs were then subjected to predict the sequence types, plasmid types, antimicrobial resistance genes, and resistance-relevant mutations in housekeeping genes using the tools available on the Center for Genomic Epidemiology website (https://www.genomicepidemiology.org/). The sequence reads obtained from the Illumina Miseq System and the Oxford Nanopore MinION sequencing platform were combined and assembled using the Unicycler assembler (34) to obtain complete genomic sequences.

### Identification of porins involved in carbapenem resistance

The OmpD sequence from *S*. Agona R21.2429 (accession number UJA67627.1) was used as a reference to search for similar porin sequences in the genomes of *S*. Agona R21.0464 (CP093402.1), *S*. Infantis R21.0914 (CP121066.1), and *S*. Typhimurium R18.0292 (CP100739.1) using the TBLASTN algorithm. Porin genes with a minimum amino acid sequence identity of 50% were further analyzed. Multiple porin sequence alignment was performed using the Clustal Omega tool, a distance matrix of the aligned sequences was generated using snp-dists (https://github.com/tseemann/snp-dists) to quantify the genetic differences among the porin sequences, and a dendrogram was constructed based on the calculated distances using the SciPy (https://scipy.org/) to show clustering patterns. Porins potentially involved in carbapenem resistance were identified by examining the porin genes in the carbapenem-resistant strains *S*. Agona R21.2430 and *S*. Infantis R13.1986, along with a previously reported *S*. Typhimurium strain (10).

## Data Availability

The complete genomic sequences of *S.* Agona strain R21.2429 and *S.* Agona strain R21.2430 were deposited in the GenBank database under the accession numbers CP090929 and CP090930, respectively. The complete sequences of the chromosome and six plasmids of *S.* Infantis strain R13.1986 can be accessed under the accession numbers CP129384-CP129390. The WGS reads for *S.* Agona R17.4368 can be found in the SRA database under the accession number SRR7458855.
